# Loss of FBXW7 and accumulation of MCL1 and PLK1 promote paclitaxel resistance in breast cancer

**DOI:** 10.18632/oncotarget.10481

**Published:** 2016-07-07

**Authors:** Jessica Gasca, Maria Luz Flores, Servando Giráldez, Manuel Ruiz-Borrego, María Tortolero, Francisco Romero, Miguel A. Japón, Carmen Sáez

**Affiliations:** ^1^ Instituto de Biomedicina de Sevilla (IBIS), Hospital Universitario Virgen del Rocío/CSIC/Universidad de Sevilla, Seville, Spain; ^2^ Department of Pathology, Hospital Universitario Virgen del Rocío, Seville, Spain; ^3^ Oncology Unit, Hospital Universitario Virgen del Rocío, Seville, Spain; ^4^ Department of Microbiology, Faculty of Biology, Universidad de Sevilla, Seville, Spain

**Keywords:** FBXW7, MCL1, PLK1, apoptosis, paclitaxel, Pathology Section

## Abstract

FBXW7 is a component of SCF (complex of SKP1, CUL1 and F-box-protein)-type ubiquitin ligases that targets several oncoproteins for ubiquitination and degradation by the proteasome. FBXW7 regulates cellular apoptosis by targeting MCL1 for ubiquitination. Recently, we identified PLK1 as a new substrate of FBXW7 modulating the intra-S-phase DNA-damage checkpoint. Taxanes are frequently used in breast cancer treatments, but the acquisition of resistance makes these treatments ineffective. We investigated the role of FBXW7 and their substrates MCL1 and PLK1 in regulating the apoptotic response to paclitaxel treatment in breast cancer cells and their expression in breast cancer tissues. Paclitaxel-sensitive MDA-MB-468 and a paclitaxel-resistant MDA-MB-468R subclone were used to study the role of FBXW7 and substrates in paclitaxel-induced apoptosis. Forced expression of FBXW7 or downregulation of MCL1 or PLK1 restored sensitivity to paclitaxel in MDA-MB-468R cells. By contrary, FBXW7-silenced MDA-MB-468 cells became resistant to paclitaxel. The expression of FBXW7 and substrates were studied in 296 invasive carcinomas by immunohistochemistry and disease-free survival was analyzed in a subset of patients treated with paclitaxel. In breast cancer tissues, loss of FBXW7 correlated with adverse prognosis markers and loss of FBXW7 and MCL1 or PLK1 accumulation were associated with diminished disease-free survival in paclitaxel-treated patients. We conclude that FBXW7 regulates the response to paclitaxel by targeting MCL1 and PLK1 in breast cancer cells and thus targeting these substrates may be a valuable adjunct for paclitaxel treatment. Also, FBXW7, MCL1 and PLK1 may be relevant predictive markers of tumor progression and response to paclitaxel treatment.

## INTRODUCTION

FBXW7 is the F-box protein of the SKP1-CUL1-FBXW7 (SCF^FBXW7^) E3 ubiquitin ligase, responsible for recruiting specific substrates for ubiquitination and degradation by the proteasome [1]. FBXW7 comprises three alternatively spliced isoforms, FBXW7 α, β and γ, of which FBXW7α is the predominant nuclear isoform in mammals [2]. Because many of the substrates that FBXW7 targets for degradation are well-known oncoproteins, such as Cyclin E, c-Myc, Aurora kinase A (AURKA) and Notch-1, FBXW7 is generally accepted as a tumor suppressor [3, 4]. FBXW7 is frequently inactivated by deletion, mutation or promoter hypermethylation in multiple neoplasias. Inactivating mutations have been most frequently found in cholangiocarcinoma, T-cell acute lymphocytic leukemia, and carcinomas of colon, endometrium and stomach [5]. In breast cancer, FBXW7 promoter hypermethylation has been identified in 51% of a series of primary tumors [6]. Many of the primary tumors with FBXW7 mutations exhibit elevated levels of Cyclin E leading to deregulation of the cell cycle [7, 8]. Loss of expression of FBXW7 has been reported in a series of breast carcinomas where Cyclin E was upregulated and associated with poor prognosis [9] but its relationship with other substrates has not been addressed. Other substrates such as c-Myc and AURKA have important roles in tumorigenesis by promoting cancer cell survival and migration [10-13] and AURKA has been found overexpressed in a series of triple negative breast carcinomas [14].

Paclitaxel is a microtubule polymerizing agent that blocks mitotic progression by activating the spindle assembly checkpoint and either causes cell death in mitosis by apoptosis or cells exit mitosis into a tetraploid G1 state, a process known as slippage [15]. Paclitaxel improves overall and disease-free survival in combination with anthracyclines or alkylating agents in metastatic and early stage breast cancer, although after the initial response, resistance to paclitaxel frequently ensues [16]. Several mechanisms for paclitaxel resistance in breast cancer have been reported including alterations in apoptotic pathways [17]. In fact, we reported that resistance to paclitaxel-induced apoptosis in MDA-MB-231breast cancer cells is related to the inability to disrupt the interaction between BCL-xL, an anti-apoptotic BCL-2 family protein, and BAK; and BCL-xL accumulation may be of prognostic value in paclitaxel-treated patients [18]. Defective degradation and accumulation of related proteins may underlie the acquisition of resistance. MCL1, a pro-survival BCL-2 family member that inhibits apoptosis by blocking the cell death mediators BAX and BAK [19], is targeted by FBXW7 for degradation upon phosphorylation by glycogen synthase kinase 3-beta (GSK3β) [20]. During anti-tubulin-induced mitotic arrest, MCL1 levels decline to potentiate cell death. Loss or inactivation of FBXW7 conferred resistance to anti-tubulin agents paclitaxel and vincristine by blocking proteasomal degradation of MCL1 [21]. We also identified this important role of MCL1 in preventing cell death during mitosis in prostate cancer cells after paclitaxel treatment [22]. Polo-like kinase 1 (PLK1) is a major mitotic kinase that plays a key role in the division of eukaryotic cells [23]. PLK1 is recruited in metaphase by several proteins such as the spindle checkpoint component BubR1 [24], which inhibits Cdc20 to prevent the activation of the anaphase promoting complex/cyclosome (APC/C). During late anaphase, PLK1 is degraded by APC/C-proteasome to properly control the exit from mitosis and cytokinesis [25]. PLK1-depleted cells are arrested in prometaphase when the spindle checkpoint is activated, but these cells fail to arrest if MAD2 or BubR1 are co-depleted [26]. PLK1 is also implicated in the replication machinery [27]. In fact, we have recently reported that PLK1 is ubiquitinated and degraded by SCF^FBXW7α^/proteasome to modulate the intra-S-phase checkpoint, where PLK1 plays an important role in the regulation of DNA replication by contributing to the formation of pre-replicative complexes [28].

Knowledge of paclitaxel resistance mechanisms will allow the identification of predictive tumor markers and therapeutic targets. Herein, we investigate the role of FBXW7 and relevant substrates in regulating apoptosis induced by paclitaxel in breast cancer cells, their expression in breast cancer tissues, and their potential predictive value in paclitaxel-treated patients.

## RESULTS

### Expression of FBXW7 and its substrates in breast cancer cell lines

**Figure 1:**
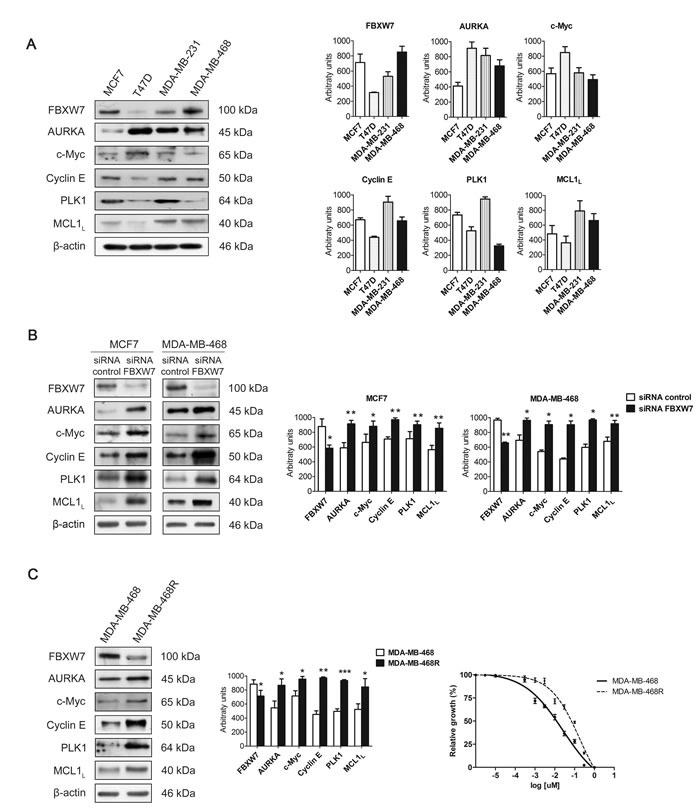
Expression of FBXW7 and its substrates in breast cancer cell lines **A.** Western blot analyses of FBXW7, AURKA, c-Myc, Cyclin E, PLK1 and MCL1 in MCF7, T47D, MDA-MB-231 and MDA-MB-468 cells in basal conditions are shown, using β-actin as loading control. Quantifications of indicated proteins are shown as histograms and data are presented as mean ± SEM. **B.** Western blot analyses of MCF7 and MDA-MB-468 cells silenced with FBXW7 siRNA and with a nontargeting control are shown; β-actin was used to ensure equal loading. Histograms show the densitometric analyses of indicated proteins. Data are presented as mean ± SEM comparing siRNA control *versus* siRNA FBXW7 in MDA-MB-468 cells and siRNA control *versus* siRNA FBXW7 in MCF7 cells. *p < 0.05, **p < 0.01 and ***p < 0.001 from Student's *t*-test. **C.** FBXW7, AURKA, c-Myc, Cyclin E, PLK1 and MCL1 were detected by Western blot in MDA-MB-468 and MDA-MB-468R cells in basal conditions. β-actin is shown as loading control. Densitometric analyses of indicated proteins are shown as histograms. Data are presented as mean ± SEM comparing MDA-MB-468 and MDA-MB-468R cells in basal conditions. *p < 0.05, **p < 0.01 and ***p < 0.001 from Student's *t*-test. IC50 curves for paclitaxel in MDA-MB-468 (solid line) and MDA-MB-468R (dotted line) cell lines are shown. Data are presented as mean ± SEM.

We selected hormone-dependent MCF7, T47D, and hormone-independent MDA-MB-231, MDA-MB-468 breast cancer cell lines to study the expression levels of FBXW7 and its substrates by western blot in basal conditions (Figure 1A). FBXW7 protein levels in MCF7, and MDA-MB-468 cells were higher than those in T47D, and MDA-MB-231 cells. Accordingly, high levels of FBXW7 correlated with low levels of its substrates. Similar results were also observed in BT474 and SKBR3 cells (Supplementary Figure S1). To confirm these results we silenced the expression of FBXW7 in MCF7 and MDA-MB-468 cells by using siRNA oligonucleotides. AURKA, c-Myc, Cyclin E, PLK1 and MCL1 levels all increased after FBXW7 downregulation in both cell lines (Figure 1B). MCF7 and MDA-MB-468 are sensitive to paclitaxel, so we generated paclitaxel-resistant MDA-MB-468R cells by selecting survival clones after paclitaxel treatment of the MDA-MB-468 cell line. In order to investigate the potential role of FBXW7 in paclitaxel resistance, we compared FBXW7 protein levels of parental MDA-MB-468 cells (IC50 0.026 ± 0.004 μM) with those of paclitaxel-resistant MDA-MB-468R cells (IC50 0.183 ± 0.002 μM) (Figure 1C). In basal conditions, paclitaxel-resistant MDA-MB-468R cells showed lower levels of FBXW7 and concordantly higher levels of AURKA, c-Myc, Cyclin E, PLK1 and MCL1 than paclitaxel-sensitive MDA-MB-468 cells (Figure 1C), suggesting the involvement of FBXW7 in paclitaxel resistance.

### Paclitaxel-induced apoptosis and cell cycle analysis of MDA-MB-468 and MDA-MB-468R cells

**Figure 2:**
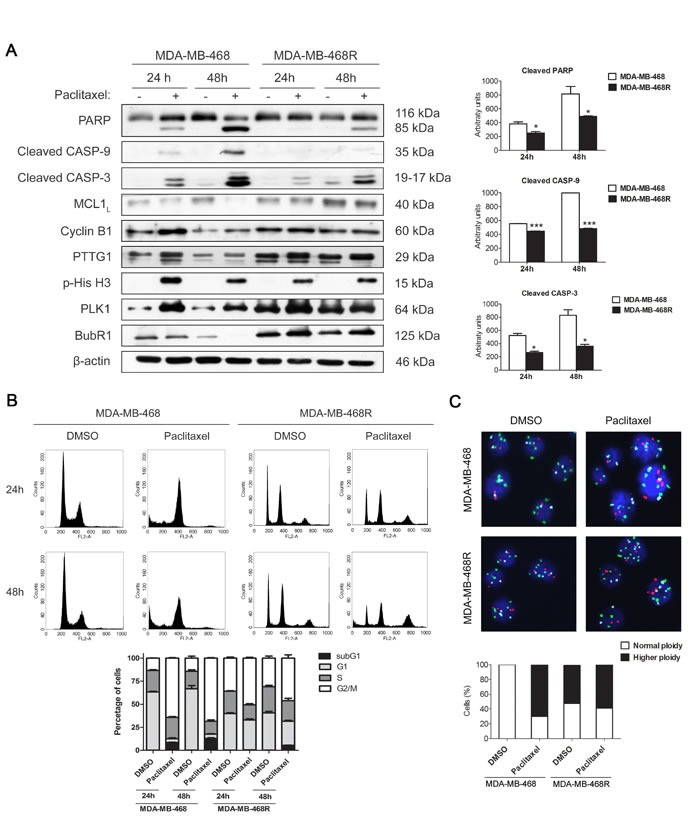
Paclitaxel-induced apoptosis and cell cycle analysis of MDA-MB-468 and MDA-MB-468R cells Cells were treated with 0.025 μM paclitaxel during 24 and 48 h. **A.** Intact and cleaved PARP, cleaved caspase-9 (CASP-9), cleaved caspase-3 (CASP-3), MCL1, Cyclin B1, PTTG1, phospho-histone H3 (p-His H3), PLK1 and BubR1 proteins were detected by western blot. β-actin is shown as loading control. Densitometric analysis of cleaved PARP, cleaved CASP-9 and cleaved CASP-3 are shown as histograms. Data from triplicate experiments are presented as mean ± SEM comparing paclitaxel-treated MDA-MB-468 *versus* paclitaxel-treated MDA-MB-468R at 24 and 48 h. *p < 0.05 and ***p < 0.001 from Student's *t*-test. **B.** Cell cycle analysis of propidium iodide-stained cells by flow cytometry. Quantification of each phase and sub-G1 cells are shown as histograms. **C.** Ploidy analysis by FISH. The number of signals per cells was determined for chromosomes 1, 11 and 17 in at least 100 cells. Representative photographs are shown. Histograms represent the percentage of cells with normal or higher ploidy for each condition.

Next, we examined paclitaxel-induced apoptosis in MDA-MB-468 and MDA-MB-468R cells by western blot (Figure 2A). Cells were treated with 0.025μM paclitaxel and collected at 24 and 48 h. Cleavage of PARP, caspase-9 and caspase-3 were induced earlier and more potently in parental MDA-MB-468 cells than in MDA-MB-468R cells. Anti-apoptotic protein MCL1 decreased after paclitaxel treatment in MDA-MB-468 cells but not in MDA-MB-468R cells, in which MCL1 remained unchanged. Paclitaxel treatment also induced the increase of Cyclin B1 and PTTG1 at 24 h in parental MDA-MB-468 cells and the later decrease of Cyclin B1, PTTG1, and BubR1 at 48 h to facilitate slippage and post-mitotic cell death. Mitotic exit is also reflected by the phospho-histone H3 (p-His H3) decrease at 48 h. In resistant MDA-MB-468R cells, Cyclin B1, PTTG1, and BubR1 increased after 24 h of paclitaxel treatment and did not fall at 48 h, p-His H3 also remained present, illustrating an association between slippage inhibition and impaired apoptosis in these cells. PLK1 needs to be degraded to allow mitotic progress and post-mitotic cell death. In sensitive MDA-MB-468 cells, PLK1 levels increased after 24 h of paclitaxel treatment and later decreased at 48 h. On the contrary, in paclitaxel treated MDA-MB-468R cells, high levels of PLK1 were still present at 48 h. Quantifications of western blot bands are shown in Supplementary Figure S2. We then analyzed the cell cycle and ploidy of paclitaxel-treated MDA-MB-468 and MDA-MB-468R cells by flow cytometry and FISH. Paclitaxel treatment induced sensitive MDA-MB-468 cells to arrest in G2/M at 24 h and increased the sub-G0/G1 apoptotic population at 48 h (Figure 2B). The cell cycle of resistant MDA-MB-468R cells was not affected after paclitaxel treatment at 24 h, but these cells showed a reduction of the G0/G1 population and fewer sub-G0/G1 apoptotic events than parental cells at 48 h. In resistant cells, FISH analysis showed higher ploidy in DMSO than in MDA-MB-468 sensitive cells. As a result of slippage, cells duplicate their DNA without cytokinesis and increase ploidy. FISH analysis showed that 60% of paclitaxel-treated MDA-MB-468 cells had higher than basal ploidy demonstrating slippage, whereas the percentage of ploidy remained unchanged in paclitaxel-treated MDA-MB-468R cells reflecting diminished slippage as a mechanism of paclitaxel resistance in these cells (Figure 2C).

### FBXW7 downregulation induces mitotic arrest and resistance to paclitaxel in MDA-MB-468 cells

**Figure 3:**
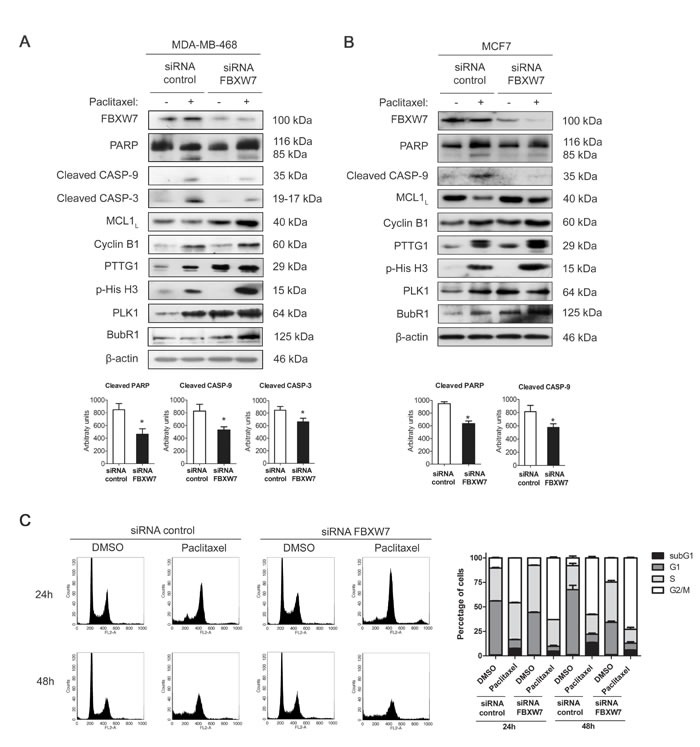
FBXW7 downregulation induces mitotic arrest and resistance to paclitaxel in MDA-MB-468 and MCF7 cells MDA-MB-468 **A.** and MCF7 **B.** cells were silenced with FBXW7 siRNA or with nontargeting control and treated with 0.025 μM paclitaxel during 24 h. Expression levels of the indicated proteins were assessed by western blot, using β-actin as loading control. Densitometric analyses of cleaved PARP, cleaved caspase-9 (CASP-9) and cleaved caspase-3 (CASP-3) are shown as histograms. Data from triplicates are presented as mean ± SEM comparing paclitaxel-treated siRNA control *versus* paclitaxel-treated siRNA FBXW7. *p < 0.05 from Student's t-test. **C.** MDA-MB-468 cell cycle analysis of propidium iodide-stained cells by flow cytometry. Quantification of each phase and sub-G1 cells are shown as histograms and data from triplicates are presented as mean ± SEM.

To gain insight into the implication of FBXW7 in paclitaxel resistance, we investigated whether the downregulation of FBXW7 influenced the paclitaxel response of sensitive MDA-MB-468 cells. As shown in Figure 3A, interfering FBXW7 diminished the sensitivity to paclitaxel treatment as demonstrated by decreased levels of cleaved PARP, caspase-9 and caspase-3. Same results were also observed in MCF7 cells (Figure 3B), so to conclude they are not cell line specific. Flow-cytometric sub-G1 analysis was performed to confirm the results in MDA-MB-468 cells (Figure 3C). The accumulation of PTTG1, p-His H3 and Cyclin B1 illustrated mitotic arrest in paclitaxel-treated FBXW7-silenced MDA-MB-468, reinforcing the influence in mitotic arrest after FBXW7 downregulation. We also confirmed increased protein levels of both FBXW7 substrates, MCL1 and PLK1. This further supports the importance of FBXW7 in achieving the complete paclitaxel response. Quantifications of western blot bands are shown in Supplementary Figure S3.

### FBXW7 overexpression restores sensitivity to paclitaxel in resistant MDA-MB-468R cells

**Figure 4:**
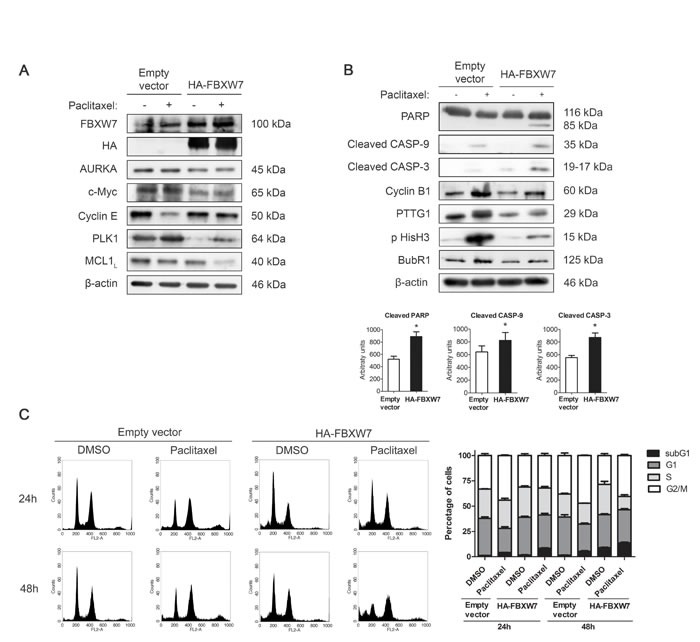
FBXW7 overexpression restores sensitivity to paclitaxel in resistant MDA-MB-468R cells Cells were transiently transfected with pCMVHA-FBXW7 or with empty vector and treated with 0.025 μM paclitaxel during 24 h. **A.** FBXW7, HA, AURKA, c-Myc, Cyclin E, PLK1 and MCL1 proteins were detected by western blot. β-actin is shown as loading control. **B.** Western blot analysis of cleaved PARP, cleaved caspase-9 (CASP-9) and cleaved caspase-3 (CASP-3) is shown; β-actin was used to ensure equal loading. Densitometric analyses of cleaved PARP, cleaved CASP-9 and cleaved CASP-3 are shown as histograms. Data from triplicates are presented as mean ± SEM comparing paclitaxel-treated empty vector *versus* paclitaxel-treated HA-FBXW7. *p < 0.05 from Student's t-test. **C.** Cell cycle analysis of propidium iodide-stained cells by flow cytometry. Quantification of each phase and sub-G1 cells are shown as histograms.

To confirm our analysis, MDA-MB-468R cells were transiently transfected with pCMVHA-FBXW7 or the empty vector and treated with 0.025 μM paclitaxel during 24 h. First, we examined the substrates of FBXW7 after transfection and treatment with paclitaxel (Figure 4A and Supplementary Figure S4A). AURKA, c-Myc, Cyclin E, PLK1, and MCL1 decreased after transfection of FBXW7 in untreated cells. After paclitaxel treatment, the changes were most remarkable in PLK1, which accumulated in mock-transfected cells and was very low in FBXW7-transfected cells, and MCL1, which dramatically reduced in the latter cell type. Next, we analyzed apoptosis and mitotic proteins in transfected cells after paclitaxel treatment (Figure 4B). Apoptosis was efficiently induced by paclitaxel in FBXW7-transfected MDA-MB-468R cells as shown by more cleavage of PARP, caspase-9 and caspase-3. Flow-cytometric sub-G1 analysis also confirmed these results (Figure 4C). Cyclin B1, PTTG1, and BubR1 levels decreased after paclitaxel treatment in FBXW7-transfected cells (Figure 4B and Supplementary Figure S4B) and showed higher ploidy after FISH analysis (Supplementary Figure S4C), indicating that these cells recover the slippage mechanism to become sensitive to paclitaxel, similarly to the parental MDA-MB-468 cells.

### MCL1 and PLK1 are major effectors of paclitaxel resistance in MDA-MB-468R cells

**Figure 5:**
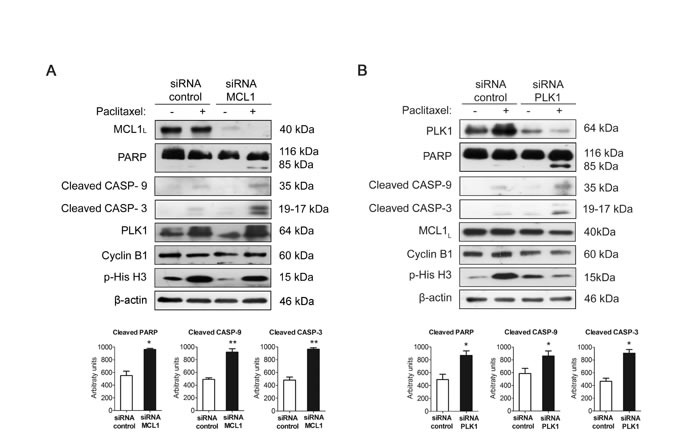
Silencing PLK1 or MCL1 restores sensitivity to paclitaxel in resistant MDA-MB-468R cells Cells were silenced **A.** with siRNA MCL1 or **B.** with siRNA PLK1 or with nontargeting controls and treated with 0.025 μM paclitaxel during 24 h. Apoptosis induction was measured by appearance of cleaved PARP, cleaved caspase-9 (CASP-9) and cleaved caspase-3 (CASP-3) by western blot. MCL1, PLK1, Cyclin B1 and p-His H3 were also assessed by western blot, using β-actin as a loading control. Densitometric analysis of cleaved PARP, cleaved caspase-9 (CASP-9) and cleaved caspase-3 (CASP-3) are shown as histograms. Data from triplicates are presented as mean ± SEM comparing paclitaxel-treated siRNA control *versus* paclitaxel-treated siRNA MCL1 or siRNA PLK1. *p < 0.05 and **p < 0.01 from Student's t-test.

We used siRNA oligonucleotides to study whether PLK1 or MCL1 downregulation may have major impact on the response to paclitaxel of resistant MDA-MB-468R cells. As expected, MCL1 downregulation increased apoptosis after 24 h of paclitaxel treatment in these cells as shown by increased levels of cleaved PARP, caspase-9 and caspase-3, therefore reverting their resistant phenotype (Figure 5A). Similarly, PLK1 downregulation was also able to promote paclitaxel-induced apoptosis efficiently in MDA-MB-468R cells (Figure 5B). Thus, we may conclude that MCL1 and PLK1 are contributors to the development of paclitaxel resistance in breast cancer cells.

To analyze the relevance of each protein in slippage, we have analyzed Cyclin B1 and p-His H3 in PLK1-silenced or MCL1-silenced MDA-MB-468R cells after treatment with DMSO or paclitaxel for 24h. In PLK1-silenced cells paclitaxel treatment decreased CyclinB1 and p-His H3 after 24h while no differences were observed in paclitaxel-treated MCL1-silenced cells (Figure 5A-5B and Supplementary Figure S5). These results may indicate that PLK1 is more relevant for the regulation of slippage than MCL1 after paclitaxel treatment.

### Loss of FBXW7 correlates with substrate accumulation and adverse prognosis markers in breast cancer tissues

**Figure 6:**
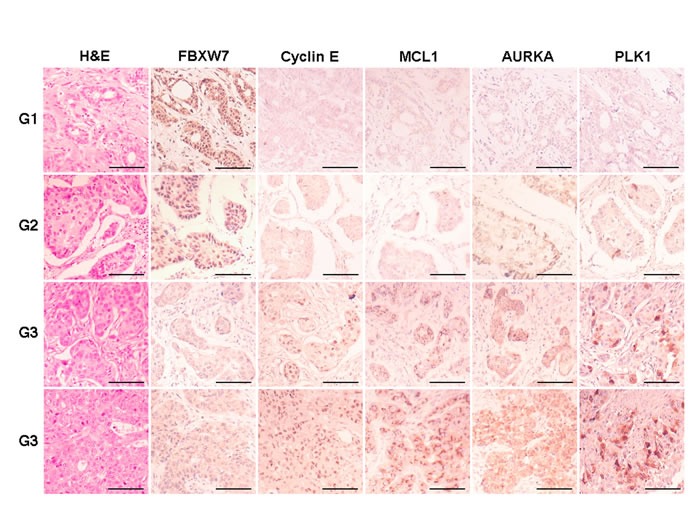
Immunohistochemical expression of FBXW7 and substrates in breast carcinomas Representative micrographs of FBXW7, Cyclin E, AURKA, MCL1 and PLK1 in breast carcinomas, tumor grade 1 to 3 (top to bottom), are shown. Scale bars, 100 μm.

To analyze FBXW7 expression and its relation to the relative abundance of substrates, and with prognostic variables in breast cancer, we performed immunohistochemistry for FBXW7, AURKA, Cyclin E, MCL1 and PLK1 in tissue microarrays containing up to 296 samples of breast carcinomas. FBXW7 was present in nuclear location of 149 tumors (50.3%) and was diminished or absent in 147 tumors (49.7%). After characterizing all samples for their FBXW7 levels, we analyzed the correlation with clinicopathological variables (Table 1). Our data show that the loss of FBXW7 expression correlated with adverse prognostic markers, e.g. high tumor grade, lack of hormone receptors or HER2 positivity. To further investigate whether FBXW7 expression correlated with its substrates in breast cancer tissues, we decided to study the protein levels of Cyclin E, MCL1, AURKA and PLK1 in these samples (Figure 6). AURKA and MCL1 were located in the cytoplasm, Cyclin E showed a nuclear pattern and PLK1 cytoplasmic and nuclear location. FBXW7 showed a statistically significant correlation with the expression of Cyclin E, MCL1 and PLK1, and the expression levels of these three proteins increased concordantly in high grade tumors.

**Table 1:** FBXW7 expression in breast carcinomas and its relationship with clinicopathological variables and substrates

FBXW7		Low		High		*p*-value^a^
		Number	(%)	Number	(%)	
Tumor	<2 cm ≥2 cm	119 28	(81.0) (19.0)	133 16	(89.3) (10.7)	0.044
Node	Negative	77	(52.4)	93	(62.4)	0.080
	Positive	70	(47.6)	56	(37.6)	
Grade	1 + 2 3	49 98	(33.3) (66.7)	107 42	(71.8) (28.2)	<0.0001
ER	Negative Positive	59 88	(40.1) (59.9)	26 123	(17.4) (82.6)	<0.0001
PR	Negative Positive	75 72	(51.0) (49.0)	39 110	(26.2) (73.8)	<0.0001
HER2	Negative Positive	114 32	(78.1) (21.9)	139 10	(93.3) (6.7)	0.0002
Ki67	Negative Positive	33 114	(22.4) (77.6)	52 97	(34.9) (65.1)	0.017
PLK1	Low High	28 119	(19.0) (81.0)	103 45	(69.6) (30.4)	< 0.0001
MCL1	Low High	44 103	(29.9) (70.1)	124 24	(83.8) (16.2)	< 0.0001
Cyclin E	Low High	40 105	(27.6) (72.4)	85 64	(57.0) (43.0)	< 0.0001
AURKA	Low High	63 84	(42.9) (57.1)	69 78	(46.9) (53.1)	0.481

ER, estrogen receptor; PR, progesterone receptor

a *p*-values were obtained from χ2 test

### Immunohistochemical expression levels of FBXW7, MCL1 and PLK1 in cancer patients are associated with paclitaxel therapy response

**Figure 7:**
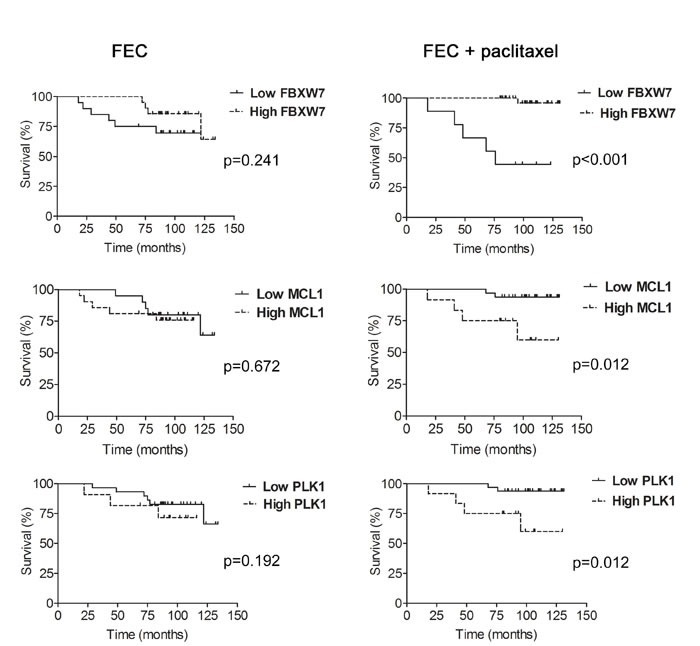
Expression levels of FBXW7, MCL1 and PLK1 and association with disease-free survival (DFS) in paclitaxel-treated patients Kaplan-Meier analysis of disease-free survival in patients treated with FEC (5-Fluorouracil, epirubicin and cyclophosphamide; left) and FECP (5-Fluorouracil, epirubicin, cyclophosphamide and paclitaxel; right) showing either high (dotted line) or low (solid line) expression of FBXW7, MCL1 and PLK1. Tick marks represent censored patients. The p values were obtained from the long-rank test of Mantel and Cox.

To assess the relationship between FBXW7, MCL1 and PLK1 expression and the response to chemotherapy in breast cancer, we performed an analysis of FBXW7, MCL1 and PLK1 expression in two groups of node-negative breast cancer patients treated with FEC (5-Fluorouracil, epirubicin, cyclophosphamide; *n* = 41) or FEC with paclitaxel (FECP; *n* = 44). Diagnostic biopsies of breast cancer patients were analyzed by immunohistochemistry for the expression of FBXW7, MCL1 and PLK1. We performed Kaplan-Meier analyses and the disease free survival was evaluated in months as the time variable. With a mean follow up time of 113.9 months after FEC treatment, disease progression was demonstrated in 30.0%, 25.0% and 20.7% patients with low FBXW7, MCL1 or PLK1 expression, respectively; and in 19%, 23.8% and 33.3% patients with high FBXW7, MCL1 or PLK1 expression. The mean time for achieving disease progression was 97.6 months, 118.9 months and 119.3 months in patients with low FBXW7, MCL1 or PLK1-expressing tumors; and 122.9 months, 97.6 months and 91.0 months in patients with high FBXW7, MCL1 or PLK1-expressing tumors. Kaplan-Meier curves were not significantly different (*p* = 0.241, *p* = 0.670 and *p* = 0.192 from Mantel and Cox, log-rank test; χ^2^ = 1.377, χ^2^ = 0.182, χ^2^ = 1.7; 1 degree of freedom) between both groups for FBXW7, MCL1 and PLK1 expression, respectively (Figure 7). In contrast, at the mean follow-up of 120.6 months post FECP treatment, disease progression was demonstrated in 55.5% patients with low FBXW7 expression, 6.25% patients with low MCL1 or PLK1 expression; and in 2.8% patients with high FBXW7 expression and in 33.3% patients with high MCL1 or PLK1 expression. The mean time for achieving disease progression was 82.5 months in patients with low FBXW7-expressing tumors, and 129.5 months in patients with high FBXW7-expressing tumors. Kaplan-Meier curves were significantly different (*p* <0.001 from Mantel and Cox, log-rank test; χ^2^ = 20.972; 1 degree of freedom) between both groups. The mean time for achieving disease progression was 127.3 months in patients with low MCL1 or PLK1-expressing tumors and 101.2 months in patients with high MCL1 or PLK1-expressing tumors. Kaplan-Meier curves were significantly different (*p* = 0.012 from Mantel and Cox, log-rank test; χ^2^ = 6.35; 1 degree of freedom) between both groups (Figure 7).

## DISCUSSION

FBXW7 acts as a tumor suppressor involved in the degradation of substrates with oncogenic activity frequently overexpressed in breast cancer such as Cyclin E, c-Myc and AURKA [14, 29-31]. It also controls degradation of MCL1 [20, 21], which has an important role in the regulation of apoptosis, and PLK1 [28], a protein kinase involved in essential events of the cell cycle [25, 32]. Treatment with taxanes improves survival of node-negative breast cancer patients [33], but acquisition of resistance can cause this treatment to lose effectivity [15, 17]. We sought to investigate the role of FBXW7 in the response to paclitaxel in breast cancer cells and in patients with node-negative disease. Commonly used breast cancer cell lines have different responses to paclitaxel [18]. We chose basal-like phenotype, paclitaxel-sensitive MDA-MB-468 cells that express abundant FBXW7 to study the role of this protein in mitotic arrest and apoptosis induced by paclitaxel, with special focus on the regulation of MCL1 and PLK1. We observed that paclitaxel-resistant subclonal MDA-MB-468R cells had lower levels of FBXW7 and accumulated PLK1 and MCL1 to impair mitosis progress and apoptosis after paclitaxel treatment. The silencing of FBXW7 in sensitive MDA-MB-468 and MCF7 cells induced the accumulation of both substrates and disabled apoptosis in response to paclitaxel. This prompted us to consider FBXW7 as an important mediator of the paclitaxel response through the control of MCL1 and PLK1 degradation in breast cancer.

MCL1 is an anti-apoptotic protein involved in resistance to cancer therapies such as cisplatin in lung cancer [34] or rituximab in lymphomas [35, 36]. MCL1 is also relevant in the response to anti-mitotic agents because cell death in mitosis may depend on MCL1 phosphorylation by CDK1-Cyclin B1 complex and its subsequent degradation [37]. Wertz et al. showed that MCL1 was stabilized in FBXW7-deficient cells arrested in mitosis and that JNK/p38/CKII kinases were regulators of the MCL1 degradation. These authors also showed that sensitivity to anti-mitotic agents was regulated by FBXW7 through degradation of MCL1 in colon and ovarian cancer cells. We observed that resistant MDA-MB-468R cells, which are FBXW7-deficient, arrest in mitosis after paclitaxel treatment but MCL1 levels remain high and apoptosis diminishes. This also occurred when FBXW7 mRNA is interfered in paclitaxel-sensitive MDA-MB-468 cells. Sensitivity to paclitaxel-induced apoptosis can be restored in resistant cells by either reintroducing FBXW7 expression or interfering MCL1. Furthermore, we demonstrated that PLK1 is also involved in paclitaxel resistance because decreasing the levels of PLK1 by siRNA sensitized resistant breast cancer cells to treatment with paclitaxel. In this sense, PLK1 levels have been related to improved sensitivity to paclitaxel and trastuzumab when combined with siRNA targeting PLK1 in breast cancer cell lines in a synergistic way [38].

After anti-mitotic treatments, cells may die in mitosis, or may either undergo slippage and then die, stay in G0, or enter a new cell cycle with increased ploidy [15]. We observed that paclitaxel-sensitive MDA-MB-468 cells die after slippage while paclitaxel-resistant MDA-MB-468R cells survived, probably after slippage, and continued cycling into an aneuploid state. These results are in concordance with those of Finkin et al., who reported that FBXW7-deficient cells are more likely to escape mitosis after anti-mitotic treatments and accumulate genetic errors [39]. Whether mitotic slippage constitutes a cell survival strategy or a cell death mechanism is not yet well understood. Riffell et al. showed that reinforcing mitotic arrest with drugs that inhibit mitotic slippage can lead to increased cell survival and proliferation, whereas inducing mitotic slippage in cells treated with antimitotic drugs seems to lead to cell death [40]. We previously observed that slippage-prone PC3 cells suffered less efficient apoptosis when they were arrested in mitosis and MCL1 was not properly degraded [22]. Therefore, MCL1 degradation has great importance in the balance between mitosis and apoptosis [41]. In this sense, the control of MCL1 degradation by FBXW7 seems determinant and we observed that FBXW7 transfection in paclitaxel resistant cells decreased the levels of MCL1 and restored sensitivity to apoptosis. Also, paclitaxel-resistant cells transfected with FBXW7 were prone to mitotic slippage as noted by the downregulation of Cyclin B1, PTTG1 and BubR1, and thus they recapitulated the behavior of paclitaxel-sensitive cells.

We noticed that the paclitaxel-resistant subclone had aneuploid DNA content. This genetic instability could be related to FBXW7 loss as it contributes to the accumulation of Cyclin E, AURKA, BubR1 and PLK1. These findings are consistent with those reported by Finkin et al., where FBXW7-deficient cells showed aneuploidy associated with increased Cyclin E and AURKA, and that AURKA could mediate the induction of aneuploidy by the p53-LATS2 pathway [39]. Moreover, PLK1 contributes to SAC regulation [42], and components of the SAC, such as BubR1, are associated with chromosomal instability [43, 44]. Dysregulated activity of PLK1 results in abnormalities in microtubules and increased centrosome size and number, and both phenomena have been related to aneuploidy [45]. Therefore, FBXW7 could be contributing to increased genetic instability through the regulation of PLK1 since FBXW7-deficient cells have higher levels of PLK1.

We show that paclitaxel resistant cells are aneuploid and accumulate MCL1 and PLK1 as well as in sensitive cells silenced for FBXW7. In cells silenced for FBXW7, paclitaxel treatment arrested cells more efficiently in mitosis, thus preventing the degradation of both substrates. These results point to the involvement of FBXW7 in the control of the degradation of these substrates in breast cancer cells and in response to apoptosis in cells arrested in mitosis. PLK1 needs to be degraded by the APC/C^CDH1^-proteasome in late anaphase to adequately control the exit from mitosis and cytokinesis so that cells can enter in G1 phase [25], but as we see in Figure 3, BubR1 is increased in cells silenced for FBXW7 so the SAC remains active. This could contribute to the inactivation of APC/Cdc20, thus preventing the degradation of PTTG1, Cyclin B1 and PLK1. Elowe et al. demonstrated that diminished BubR1 contributes to cells escaping by slippage [24]. We observed a similar response in the paclitaxel sensitive cells, where BubR1 and PLK1 levels are low, and taxol treatment induced the cells to exit mitosis by slippage with subsequent apoptosis. Consistent with these data, van Vugt et al. [26] established the role of PLK1 in the control of mitosis by regulation of the spindle checkpoint. They showed that PLK1 depleted cells arrest in prometaphase with an activated spindle checkpoint, but if these cells are BubR1 co-depleted, they fail to arrest in mitosis. Accordingly, resistant cells and cells silenced for FBXW7 accumulate both proteins, particularly PLK1, an accumulation that remains after paclitaxel treatment and is accompanied by efficient mitotic arrest with less death by apoptosis.

We also looked at the relative expression of FBXW7 and its substrates in a series of breast carcinoma tissues to establish whether these proteins could be valuable prognostic markers and predict patient response to paclitaxel treatment. We observed that expression levels of FBXW7 correlated inversely with the levels of substrates in a statistically significant way with Cyclin E, PLK1 and MCL1. Ibusuki et al. previously reported that a decreased expression of FBXW7 was associated with increased levels of Cyclin E and c-Myc in a group of patients with breast carcinoma of a more aggressive tumor stage [9]. We see that loss of nuclear FBXW7 associates significantly with higher tumor size and grade and with HER2-positive and triple negative phenotypes. Our study also shows that loss of FBXW7 and increased levels of MCL1 and PLK1 are associated with lower disease-free survival in patients whose treatment includes paclitaxel. We conclude that FBXW7 can be a marker of prognostic value and that the patient's response to paclitaxel is most effective in slowing disease progression when FBXW7 is present and MCL1 and PLK1 do not accumulate.

In summary, we show that FBXW7 plays a prominent role in paclitaxel-induced apoptosis by regulating MCL1 and PLK1 levels in breast cancer, as resistant cells loose FBXW7 and accumulate these substrates. Loss of FBXW7 is associated with adverse prognostic factors such as tumor grade or non-luminal phenotypes and FBXW7, MCL1 and PLK1 levels may have predictive value in patients treated with paclitaxel. Reintroducing FBXW7 activity or interfering MCL1 and PLK1 restores paclitaxel sensitivity in resistant cells and thus, targeting these substrates may be a valuable adjunct to paclitaxel for the treatment of breast cancer. Notably, for the first time, we observed a direct association between FBXW7 and PLK1 expression in breast cancer tissues.

## MATERIALS AND METHODS

### Cell culture

MCF7, T47D, MDA-MB-231, MDA-MB-468, SKBR3 and BT474 breast cancer cell lines from the Interlab Cell Line Collection (Genoa, Italy) were routinely grown in RPMI 1640 supplemented with 10% fetal bovine serum, 50 U/mL penicillin, 50 μg/mL streptomycin, 10 mmol/L HEPES buffer and 1 mM glutamine in a 37ºC humidified incubator under 5% CO_2_. Subconfluent cell cultures were harvested by trypsinization. All the experiments were performed using cells that had not exceeded the first ten passages after receipt of the initial vial. Stock solution of paclitaxel (Calbiochem) was prepared at 10 mM in dimethyl sulfoxide (DMSO, Sigma) and stored frozen. In all the experiments, DMSO was added to untreated cells. The paclitaxel-resistant MDA-MB-468R cell line was generated in our laboratory as follows. Parental MDA-MB-468 cells were treated with 4 nM paclitaxel for 2 months; then, the concentration of the drug in the medium was reduced by half. The surviving cells were maintained with 1 nM paclitaxel for 1 month and finally, the drug was withdrawn from the medium. The surviving cells were again subjected to the same treatment and the resulting paclitaxel-resistant cells were used for the assays.

### Small interfering RNA (siRNA) and plasmid transfections

Validated pools of FBXW7α-, MCL1-, PLK1-siRNA and non-targeting siRNA as negative control were obtained from GE Dharmacon (ON-TARGETplus SMART pools L-004264, L-004501, L-003290, and D-001810). Transfections were carried out using DharmaFECT 2 reagent (GE Dharmacon) according to manufacturer's instructions. All siRNA pools were used at 50 nM. Cells were subjected to different treatments 24 h after silencing. Transient transfections of pCMVHA and pCMVHA-FBXW7 plasmids [28] were carried out using FuGENE reagent (Promega) according to manufacturer's instructions. Cells were subjected to different treatments 24 h after transfection.

### Antibodies

The antibodies and dilutions used for western blots were as follows: rabbit polyclonal anti-FBXW7 (1:500) and anti-AURKA (1:2,000) from Novus; rabbit polyclonal anti-MCL1 (S-19, 1:1,000), anti-Cyclin B1 (1:1,000), anti-phospho-histone H3 (Ser10, 1:500) and mouse monoclonal anti-PTTG1 (1:1,000) from Santa Cruz; mouse monoclonal anti-β-actin (Ac15, 1:20,000) and rabbit polyclonal anti-c-Myc (1:1,000) from Sigma; rabbit polyclonal anti-cleaved caspase-9 (Asp 315, 1:500), anti-cleaved caspase-3 (Asp 175, 1:500) from Cell Signaling; rabbit polyclonal anti-BubR1 (1:3,000) from Bethyl; mouse monoclonal anti-PARP (1:500) from BD Bioscience; mouse monoclonal anti-Cyclin E (1:500) from Monosan; mouse monoclonal anti-PLK1 (1:10,000) from Millipore, and rat monoclonal anti-HA-peroxidase (50 mU/mL) from Roche. For immunohistochemical analyses, anti- FBXW7 (1:2,000), anti- Cyclin E (1:100), anti- MCL1 (1:1,250), anti-AURKA (1:700), and anti-PLK1 (1:1,000) were used.

### Western blotting

Cells were lysed in Nonidet P-40 (NP40) lysis buffer [10 mM Tris-HCl (pH 7.5), 150 mM NaCl, 10% glycerol, and 1% NP40]. Twenty micrograms of total protein, as determined by the BCA protein assay kit (Pierce), were separated by SDS-PAGE on 8% polyacrylamide gels and electroblotted onto nitrocellulose membranes (GE Healthcare). Membranes were stained with Ponceau S to ensure that protein amounts were comparable. For immunodetection, blots were blocked in 1% blocking reagent (Roche) in 0.05% Tween 20-phosphate buffered saline (PBS) for 1 hour and incubated with primary antibody diluted in blocking buffer overnight at 4ºC. Blots were then washed in 0.05% Tween 20-PBS and incubated with either goat anti-mouse (1:20,000; GE Healthcare) or goat anti-rabbit (1:20,000; GE Healthcare) peroxidase-labeled antibodies in blocking buffer for 1 hour. The enhanced chemoluminescent system was applied according to the manufacturer's protocol (GE Healthcare). The experiments were performed at least three times and densitometric analysis was performed using ImageJ software (http://imagej.nih.gov/ij/). Arbitrary densitometric units of the proteins of interest were corrected for those of β-actin. Data comparing differences between the two conditions were statistically analyzed, when indicated, using paired Student's *t*-test. Statistical analyses were performed with Prism 4.0 software (GraphPad). Differences were considered as significant when *p* < 0.05.

### Flow cytometric analysis of cell cycle

Cells were trypsinized and fixed in 70% ethanol. Propidium iodide staining of nuclei was performed with a CycleTest Plus DNA reagent kit (BD Biosciences), and the DNA content was measured with a FACScan instrument (BD Biosciences). Data were acquired with CellQuest Pro software (BD Biosciences).

### Fluorescence *in situ* hybridization (FISH)

Cultured cells were imprinted onto silanized slides, fixed in ice-cold methanol/glacial acetic acid (3:1) for 10 min and air-dried. Then, slides were immersed in a 2x SSC/0.3% NP40 solution at 37ºC during 30 min, dehydrated in an increasing series of graded ethanols and air-dried. Cellular DNA and centromeric probes for chromosomes 8 (Spectrum red), 11 (Spectrum green) and 17 (Spectrum aqua) from Vysis were co-denatured at 72ºC for 5 min and hybridized at 37ºC overnight in a humid chamber (Thermomixer, Eppendorf). After hybridization, slides were washed in 2x SSC/0.3% NP40 solution at 72ºC for 5 min, counterstained, mounted with DAPI/antifade solution (Vysis), and visualized under fluorescence microscopy equipped with appropriate filter sets and a digital camera (Leica). At least one hundred cells were counted to calculate the percentage of cells with normal ploidy and higher ploidy in each condition.

### Cytotoxicity assay

6 x 10^3^ cells/well in exponential cell growth were plated in 96-well plates (Nunc). Following cell adherence (growth for 24 h), experimental medium containing paclitaxel was added to triplicate wells, and serial dilutions were performed to span the dose range suitable for the analysis. Cells were exposed to the drug for 72 h and a cell viability assay was performed using alamarBlue Cell Viability Assay Reagent (Thermo Scientific) according to manufacturer's instructions. Fluorescence was measured with excitation wavelength at 545 nm and emission wavelength at 590 nm with FLx800 Microplate Fluorescence Reader (Bio-Tek). Data were normalized to vehicle treatment, and the half inhibitory concentrations (IC50) were calculated using OriginPro 8 and Prism 4.0 software (GraphPad). The experiments were performed at least three times.

### Immunohistochemical staining and scoring

Formalin-fixed, paraffin-embedded tissue blocks from 296 patients with invasive breast carcinoma were selected to build up tissue microarrays with 1 mm cores. The study was approved by the Ethical Committee of the hospital. Five μm tissue sections were dewaxed, rehydrated, and immersed in 3% H_2_O_2_ aqueous solution for 30 minutes to exhaust endogenous peroxidase. Heat-induced epitope retrieval was performed with 1 mM EDTA (pH 9.0) in a microwave oven. Sections were incubated overnight at 4ºC with the primary antibodies. Peroxidase-labeled secondary antibodies and 3,3′-diaminobenzidine were applied according to manufacturer's protocol (EnVision, Dako). Slides were then counterstained with hematoxylin and mounted. Sections where the primary antibody was omitted were used as negative controls. Immunostains were scored as low (<25%) or high expression (≥25%) according to the extent of positive cells. Correlations between proteins and clinicopathological variables were analysed by χ^2^ test using Prism 4.0 software (GraphPad). Differences were considered as significant when *p* < 0.05. The association of FBXW7, MCL1 and PLK1with paclitaxel therapy response was analyzed by the method of Kaplan-Meier.

## SUPPLEMENTARY MATERIAL FIGURES



## Abbreviations

DAPI: 4′,6-Diamidino-2-phenyl-indole
DMSO: Dimethyl sulfoxide
NP40: Nonidet-P40
PARP: Poly (ADP-ribose) polymerase.
