# Supplementary material for: Loss of FBXW7 and accumulation of MCL1 and PLK1 promote paclitaxel resistance in breast cancer
Source: Oncotarget. 2016 Jul 7;7(33):52751–65. doi: 10.18632/oncotarget.10481 (PMC5288146; doi:10.18632/oncotarget.10481)
Supplement: Supplementary file 1 [file oncotarget-07-52751-s001.pdf]

Loss of fbxw7 and accumulation of mcl1 and plk1 promote paclitaxel resistance in breast cancer

Supplementary Material

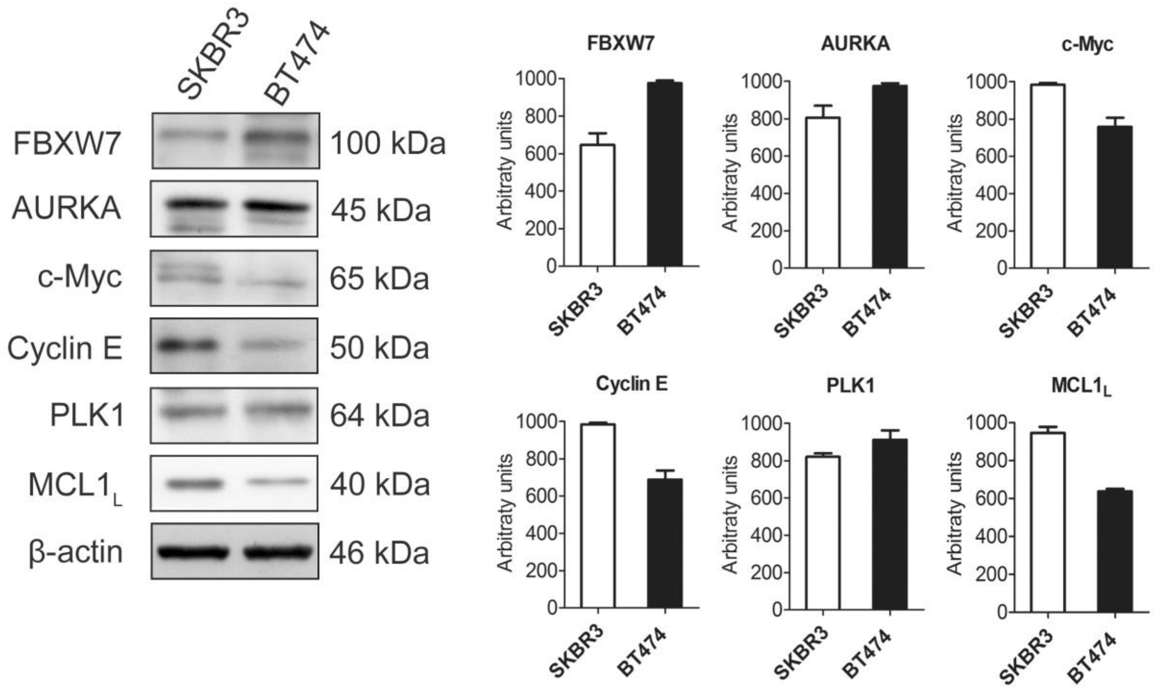

Supplementary Figure S1. Western blot analysis of FBXW7, AURKA, c-Myc, Cyclin E, PLK1 and MCL1 in SKBR3 and BT474 cell lines in basal conditions are shown, using β-actin as loading control. Experiments were performed at least three times. Histograms show the quantification of indicated proteins.

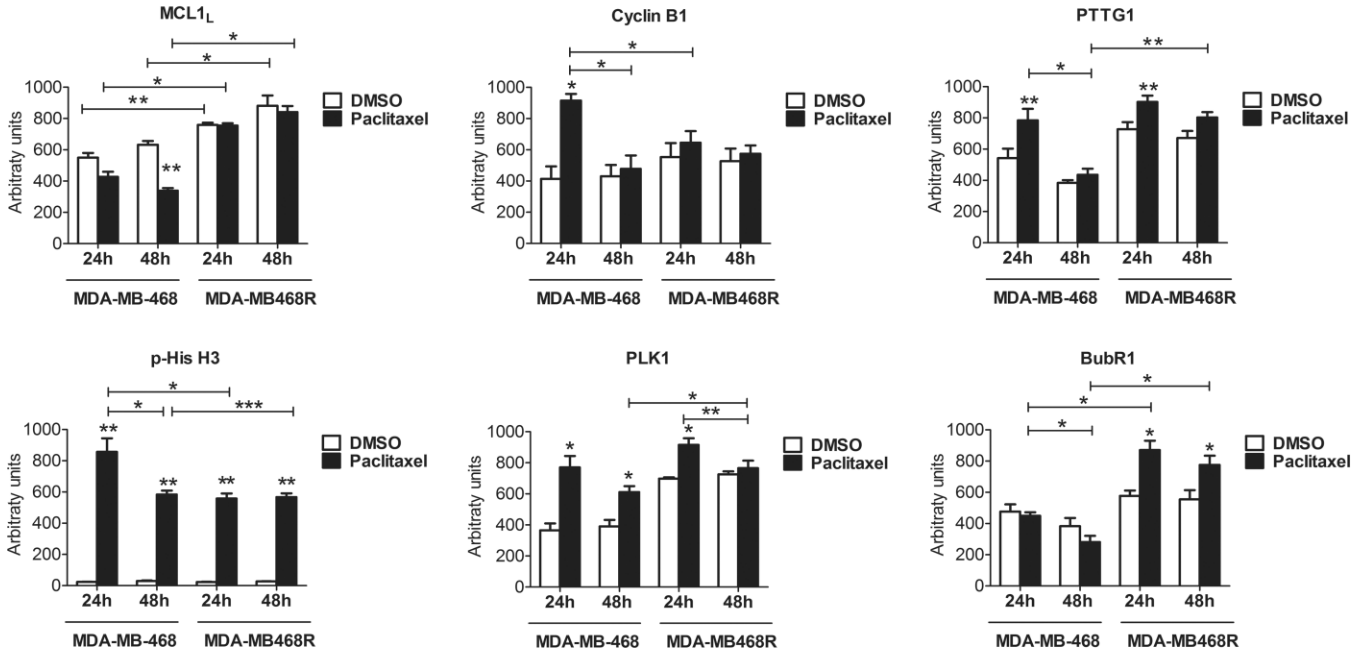

Supplementary Figure S2. Densitometric analysis of MCL1, Cyclin B1, PTTG1, phospho-histone H3 (p-His H3), PLK1 and BubR1 in MDA-MB468 and MDA-MB468R comparing in both cell lines DMSO 24 h versus paclitaxel 24 h, DMSO 48 h versus paclitaxel 48 h, and paclitaxel 24 h versus paclitaxel 48 h. Comparison of DMSO-treated MDA-MB-468 versus DMSO-treated MDA-MB-468R at 24 and at 48 h and paclitaxel-treated MDA-MB-468 versus paclitaxel-treated MDA-MB-468R at 24 and at 48h are also shown. Data from triplicates are presented as mean ± SEM comparing MDA-MB-468 and MDA-MB-468R cells. *p<0.05, **p<0.01 and ***p<0.001 from Student's t-test.

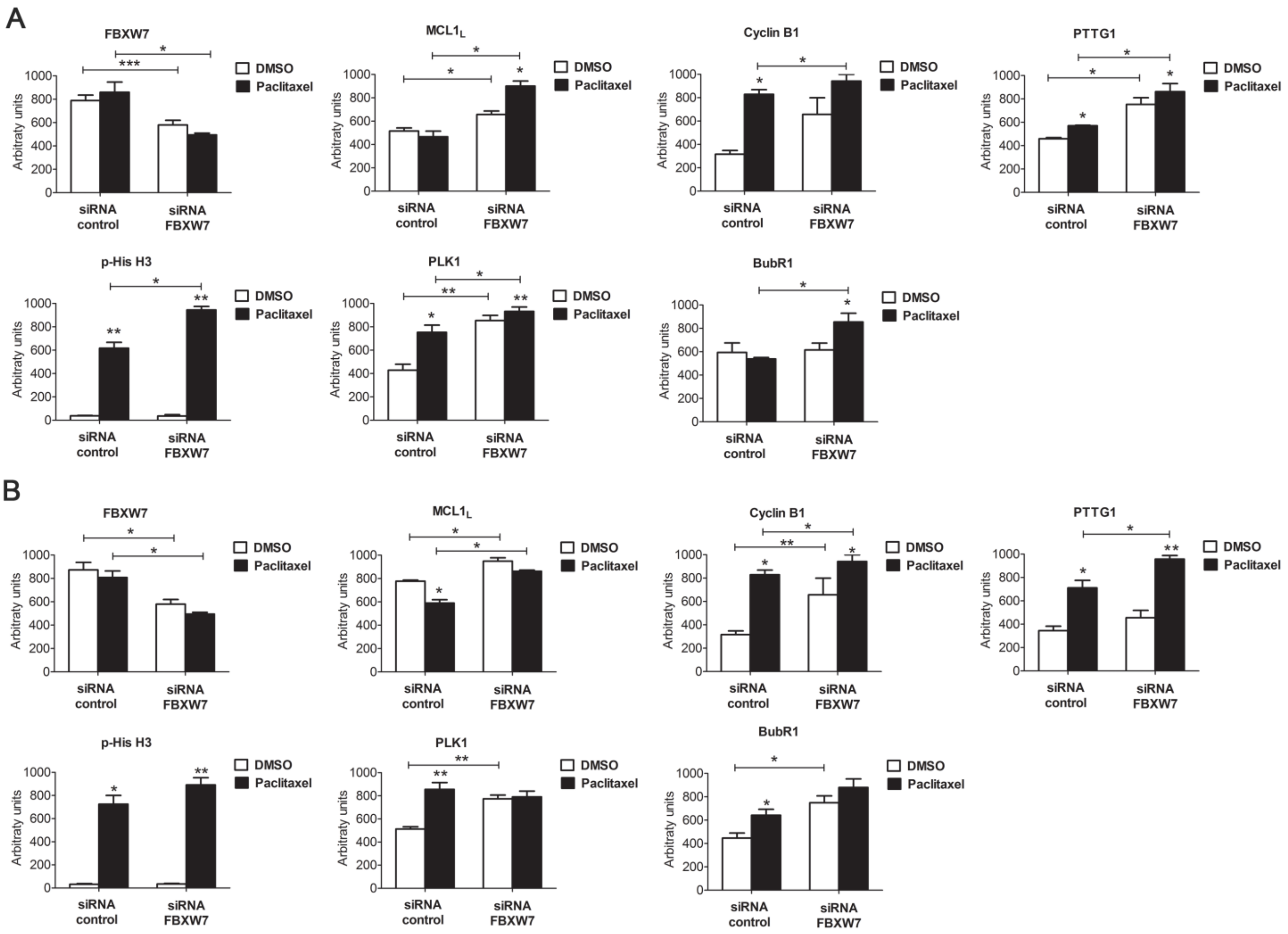

Supplementary Figure S3. (A) Densitometric analysis of FBXW7, MCL1, CyclinB1, PTTG1, phospho-histone H3 (p-His H3), PLK1 and BubR1 in MDA-MB-468 are shown as histograms. Data from triplicates are presented as mean ± SEM comparing DMSO-treated siRNA control versus paclitaxel-treated siRNA control, DMSO-treated siRNA FBXW7 versus paclitaxel-treated siRNA FBXW7, DMSO-treated siRNA control versus DMSO-treated siRNA FBXW7, paclitaxel-treated siRNA control versus paclitaxel-treated siRNA FBXW7. *p<0.05, **p<0.01 and ***p<0.001 from Student's t-test *p<0.05 from Student's t-test.
(B) Densitometric analysis of FBXW7, MCL1, CyclinB1, PTTG1, phospho-histone H3 (p-His H3), PLK1 and BubR1 in MCF7 are shown as histograms. Data from triplicates are presented as mean ± SEM comparing DMSO-treated siRNA control versus paclitaxel-treated siRNA control, DMSO-treated siRNA FBXW7 versus paclitaxel-treated siRNA FBXW7, DMSO-treated siRNA control versus DMSO-treated siRNA FBXW7, paclitaxel-treated siRNA control versus paclitaxel-treated siRNA FBXW7. *p<0.05 and **p<0.01 from Student's t-test.

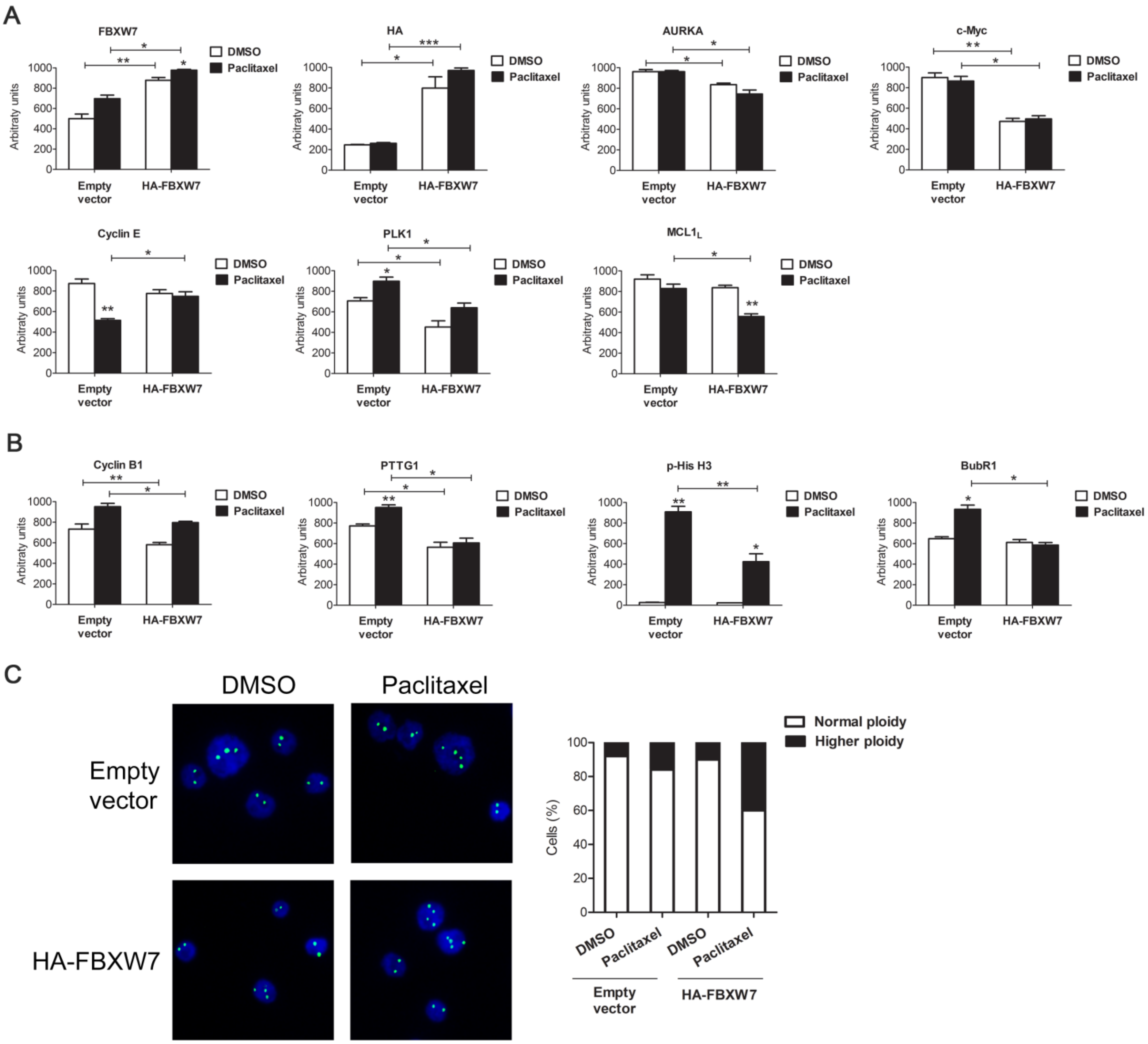

Supplementary Figure S4. (A) Densitometric analysis of FBXW7, HA, AURKA, c-Myc, Cyclin E, PLK1 and MCL1 in MDA-MB-468R are shown as histograms comparing DMSO-treated empty vector versus paclitaxel-treated empty vector, DMSO-treated HA-FBXW7 versus paclitaxel-treated HA-FBXW7, DMSO-treated empty vector versus DMSO-treated HA-FBXW7, paclitaxel-treated empty vector versus paclitaxel-treated HA- FBXW7. Data from triplicate are presented as mean ± SEM. *p<0.05, **p<0.01 and ***p<0.001 from Student's t-test. **(B)** Densitometric analysis of Cyclin B1, PTTG1, phospho-histone H3 (p-His H3) and BubR1 in MDA-MB-468R are shown as histogram. Data from triplicate are presented as mean ± SEM comparing DMSO-treated empty vector versus paclitaxel-treated empty vector, DMSO-treated HA-FBXW7 versus paclitaxel-treated HA-FBXW7, DMSO-treated empty vector versus DMSO-treated HA-FBXW7, paclitaxel-treated empty vector versus paclitaxel-treated HA- FBXW7. *p<0.05, **p<0.01 and ***p<0.001 from Student's t-test. **(C)** Ploidy analysis by FISH. The number of signals per cells was determined for chromosome 17 (spectrum green) in at least 100 cells. DNA was stained with DAPI (blue). Representative photographs are shown. Histograms represent the percentage of cells with normal or higher ploidy for each condition.

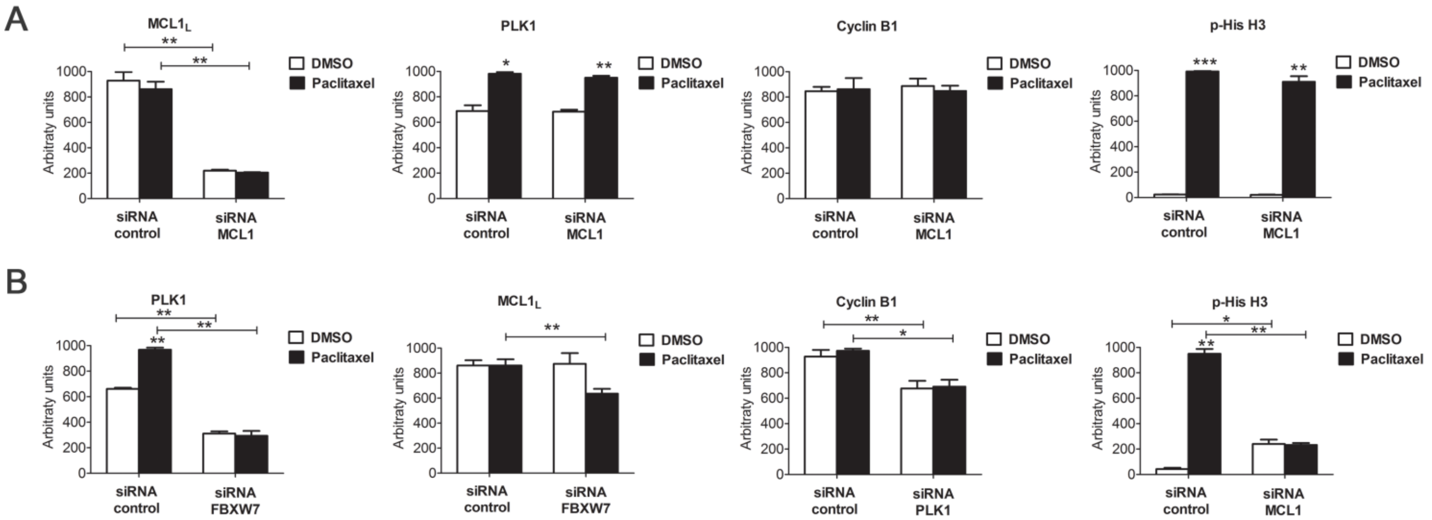

Supplementary Figure S5. (A) Densitometric analysis of MCL1, PLK1, Cyclin B1 and phospho-histone H3 (p-His H3) in MDA-MB-468R are shown as histograms comparing DMSO-treated siRNA control versus paclitaxel-treated siRNA control, DMSO-treated siRNA MCL1 versus paclitaxel-treated siRNA MCL1, DMSO-treated siRNA control versus DMSO-treated siRNA MCL1, paclitaxel-treated siRNA control versus paclitaxel-treated siRNA MCL1. Data from triplicates are presented as mean ± SEM. *p<0.05, **p<0.01 and ***p<0.001 from Student's t-test. **(B)** Densitometric analysis of PLK1, MCL1, Cyclin B1 and phospho-histone H3 (p-His H3) in MDA-MB-468R are shown as histograms comparing DMSO-treated siRNA control versus paclitaxel-treated siRNA control, DMSO-treated siRNA PLK1 versus paclitaxel-treated siRNA PLK1, DMSO-treated siRNA control versus DMSO-treated siRNA PLK1, paclitaxel-treated siRNA control versus paclitaxel-treated siRNA PLK1. Data from triplicates are presented as mean ± SEM. *p<0.05, **p<0.01 and ***p<0.001 from Student's t-test.
